# Profiling the heterogeneity of microbial populations and communities at the single‐cell level

**DOI:** 10.1002/mlf2.70047

**Published:** 2025-10-27

**Authors:** Lu Wu, Wenlong Zuo, Zhaohui Cao, Zepeng Qu, Lei Dai

**Affiliations:** ^1^ State Key Laboratory of Quantitative Synthetic Biology, Shenzhen Institute of Synthetic Biology, Shenzhen Institutes of Advanced Technology, Chinese Academy of Sciences Shenzhen China; ^2^ State Key Laboratory of Vaccines for Infectious Diseases, Xiang An Biomedicine Laboratory, School of Public Health, Xiamen University Xiamen China; ^3^ University of Chinese Academy of Sciences Beijing China

**Keywords:** heterogeneity, microbial populations, single‐cell level, spatial

## Abstract

Recent advancements in single‐cell genomic and transcriptomic sequencing, in situ sequencing, and molecular imaging‐based technologies have facilitated the examination of heterogeneity within microbial communities at the single‐cell level. These cutting‐edge methodologies permit the capture of phenotypic and genotypic heterogeneity, as well as the spatial organization within microbial communities. This enables in‐depth investigation into microbial dark matter, the evaluation of microbial responses to perturbations, and a comprehensive exploration of spatial functions involved in community assembly and social interactions within microbial communities. These interactions include inter‐microbial relationships, bacteria–phage interactions, and host–microbe interactions. Here, we highlight the key technological breakthroughs achieved, elucidating the perspectives from which these technologies enable us to interpret microbial heterogeneity at the single‐cell level. Additionally, we critically examine the limitations associated with these technologies. Furthermore, we explore how these methods could be combined and also their applications in future studies. The integration of these approaches holds great promise for increasing our understanding of the organization and function of microbes in complex ecosystems.

## INTRODUCTION

Microorganisms play important roles in almost all types of ecosystems. Their diversity, metabolic capabilities, and interactions make them essential components of ecological communities and vital for the functioning and sustainability of ecosystems. The microbial community is not a homogeneous blend but has a structured organization shaped by internal interactions, biochemical environments, and association with multicellular hosts, such as plants, animals, and humans. Furthermore, microbes within a community display phenotypic and genotypic heterogeneity that is influenced by their micro‐habitats and evolutionary processes, such as genetic drift and horizontal gene transfer. Therefore, characterizing the spatial, phenotypic, and genotypic heterogeneity of the microbial community is of great importance for understanding the assembly and ecological function of microorganisms.

In the past two decades, researchers have developed numerous strategies to characterize the heterogeneity within microbial communities, allowing for a more comprehensive analysis. These strategies have provided valuable insights into the heterogeneities, interactions, and functional potentials of microorganisms within their ecosystems. The advent of high‐throughput sequencing has catalyzed a paradigm shift in microbiome research, yielding unprecedented insights into microbial influences on ecological dynamics, human health, agriculture, and environmental science. However, traditional sequencing approaches often provide limited information about the heterogeneity present within microbial communities. Single‐cell sequencing, as a new emerging approach to address microbial community heterogeneity, enables the analysis of individual microbial cells, providing a high‐resolution view of the genetic and functional diversity within a community through single‐cell genomics and transcriptomics. This approach allows for the identification of rare or novel microbial taxa, as well as the detection of transcriptional variations and metabolic activities among individual cells; however, it loses spatial information. In recent years, sequencing combined with spatially resolved techniques has been developed to obtain spatial information. Such techniques as in situ genome and RNA sequencing (IGS^1^ and ISSeq^2^) techniques allow for direct sequencing in situ, also providing a highly resolved view of spatial heterogeneity. Coupled with next‐generation sequencing (NGS)^3^, ^4^, ^5^, ^6^, capture‐based technologies that use slides that contain spots or beads with barcoded oligonucleotides for DNA/mRNA capture also enable the understanding of host–microbe interactions in health and disease. Laser‐capture microdissection (LCM) is also applicable when coupled with sequencing to achieve a precise analysis of cell population heterogeneity with spatial information^7^, ^8^.

Molecular imaging represents another powerful set of tools to uncover the heterogeneity within microbial communities, which could provide not only genotypic and phenotypic heterogeneity but also detailed spatial information. Molecular imaging technologies, which visualize various molecules within microbes such as DNA, RNA, proteins, lipids, and metabolites, play a pivotal role in uncovering the heterogeneity within microbial communities. These technologies, whether using labeled or unlabeled molecules, include notable imaging techniques such as optical imaging, acoustic imaging, magnetic resonance imaging (MRI), and nuclear medicine imaging. Among these techniques, three methods are particularly prominent: fluorescence in situ hybridization (FISH), which involves staining nucleic acids; metabolic labeling‐based imaging of metabolites such as amino acids, proteins, and sugars; and Raman spectroscopy, a labeling‐free technique. These approaches are frequently used due to their effectiveness in studying the composition and dynamics of microbial ecosystems.

**Figure 1:**
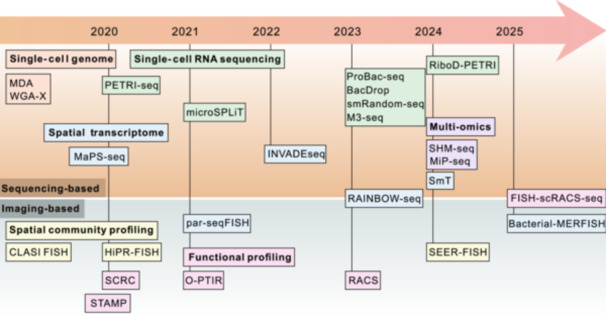
Emerging approaches for profiling microbial population heterogeneity at the single‐cell level. The methods are broadly categorized into two groups: sequencing‐ and imaging‐based approaches. Some techniques, such as RAINBOW‐seq and FISH‐scRACS‐seq, represent hybrid strategies that combine both modalities. The purpose of each method is indicated by the color of the text box, corresponding to specific goals: single‐cell genome, single‐cell RNA sequencing, spatial community profiling, spatial transcriptome, functional profiling, or multi‐omic integration. BacDrop, bacterial droplet‐based single‐cell RNA‐seq; Bacterial‐MERFISH, bacterial multiplexed error‐robust FISH; CLASI FISH, combined labeling and spectra Identification FISH; FISH, fluorescence in situ hybridization; FISH‐scRACS‐seq, FISH guided scRACS‐seq; HiPR‐FISH, high‐phylogenetic‐resolution microbiome mapping by FISH; INVADEseq, invasion–adhesion‐directed expression sequencing; M3‐seq, single‐cell massively‐parallel multiplexed microbial sequencing; MaPS‐seq, metagenomic plot sampling by sequencing; MDA, multiple displacement amplification; microSPLiT, microbial split‐pool ligation ligation transcriptomics; MiP‐seq, multi‐omics in situ pairwise sequencing; O‐PTIR, optical photothermal infrared spectroscopy; par‐seqFISH, parallel sequential FISH; PETRI‐seq, prokaryotic expression profiling by tagging RNA in situ and sequencing; ProBac‐seq, probe‐based bacterial sequencing; RAINBOW‐seq, labeling of biofilms in a rainbow‐like pattern; RACS, Raman‐activated cell sorting; RiboD‐PETRI, rRNA‐derived cDNA depletion strategy based PERTI; SEER‐FISH, sequential error‐robust FISH; SHM‐seq, spatial host–microbiome sequencing; smRandom‐seq, Droplet‐based high‐throughput single‐microbe RNA‐seq assay; SmT, spatial metatranscriptomics; STAMP, sequential tagging with fluorescent D‐amino acid‐based metabolic probes.

This review focuses on the latest approaches that enhance our characterization of the heterogeneity of microbial population and communities at the single‐cell level (Figure 1, Table 1). We will primarily explore the methods that enable the profiling of spatial, phenotypic, and genotypic heterogeneity within microbial communities. We will also discuss how these methods could be combined and also their applications in future studies. Utilizing these approaches could enhance our understanding of the organization and function of microbes in complex ecosystems, such as soil, plant rhizosphere, and animal gut.

**Table 1:** Summary of advanced methodologies for microbial single‐cell and spatial analyses.

Category	Method/Technology	Advantages	Limitations
Single‐cell genomics	scWGA (e.g., MDA)	− Culture‐independent profiling of unculturable microbes − Capturing strain‐level genomic heterogeneity (HGT, phage interactions)	− Amplification bias (e.g., GC bias) − High equipment cost (microfluidics/FACS) − Uneven genome coverage
	WGA‐X (thermostable phi29)	− Improved coverage for high‐GC genomes	− Residual amplification biases
Single‐cell transcriptomics	MATQ‐seq	− Random hexamer priming for bacterial mRNA (no poly(A) dependency) − Detection of ~200 transcripts/cell	− Low throughput (limited cells per run)
	PETRI‐seq/microSPLiT	− High throughput (10,000+ cells) − Compatible with Gram^+^/Gram^−^ bacteria	− Requiring multi‐round indexing, complex workflow
	RiboD‐PETRI	− Cost‐effective and equipment‐free	− Probe limited to species‐specific rRNA depletion
	ProBac‐seq/BacDrop	− 10× Genomics compatibility − Probe‐based rRNA reduction (ProBac‐seq)	− Probe design limited to known genomes (ProBac‐seq) − Low mRNA capture (BacDrop: <100 mRNAs/cell)
	smRandom‐seq	− Random primers for in situ cDNA generation − CRISPR‐based rRNA depletion	− sgRNAs were designed to target specific sequences
	M3‐seq	− Post‐amplification rRNA depletion − Multiplexed species/conditions	− Computational complexity
Sequencing‐based spatial technologies	MaPS‐seq	− Micron‐scale spatial co‐localization analysis − Integration of host transcriptomics and microbial taxonomy	− Cryofracture may disrupt native structures
	INVADEseq/SHM‐seq	− In situ host–microbe interaction mapping (e.g., tumors) − Multi‐omics (transcriptome + 16S)	− Resolution limit (~55 µm) − Restricted to host tissues
	SmT	− Simultaneous host transcriptome and microbial functional profiling	− Technically demanding
Molecular imaging‐based technologies	CLASI‐FISH/HiPR‐FISH	− High multiplexing − Single‐cell spatial resolution	− Spectral overlap limitations − Probe design relies on known 16S/18S/ITS sequences
	SEER‐FISH/par‐seqFISH	− Error‐robust barcoding − Combination of taxonomy and gene expression	− Multiple hybridization rounds, time‐consuming
	Bacterial‐MERFISH	− Enabling ~1000‐fold volumetric expansion, resolving high RNA density − Subcellular resolution reveals transcriptome spatial organization	− Probe design requiring prior knowledge of target genes − Lower throughput and complex sample preparation
	RAINBOW‐seq	− Combination of growth‐based labeling, FACS, and RNA‐seq for spatially resolved transcriptomics − Linking cellular states to spatial context with high molecular resolution	− Requiring cell sorting, potentially losing native spatial information − Dependent on metabolic labeling efficiency
	Metabolic Labeling (STAMP)	− In vivo tracking of microbial colonization/metabolism − 3D imaging with FISH	− Requiring chemical precursors, potential metabolic interference
	Single‐Cell Raman spectroscopy (SCRS)	− Label‐free metabolic fingerprinting − Coupling with sorting/sequencing (scRACS‐Seq)	− Peak interpretation requiring machine learning − Limited reference databases
	O‐PTIR Spectroscopy	− Label‐free detection of biomolecules (e.g., polyhydroxybutyrate (PHB)) − Semi‐quantitative single‐cell metabolism	− Specialized equipment, low accessibility
	Live Imaging (Bioluminescence/near‐infrared (NIR))	− Real‐time microbial activity monitoring (e.g., colonization) − Noninvasive	− Requiring genetic reporters/exogenous probes
	NanoSIMS	− Isotope tracing for element flow (e.g., C/N) − Subcellular resolution	− Complex sample preparation, expensive

## HIGH‐THROUGHPUT SEQUENCING‐BASED TECHNOLOGIES

The wide application of next‐generation sequencing (NGS) and third‐generation sequencing technologies has revolutionized the microbial community studies. These sequencing‐based methods, such as metagenomics and meta‐transcriptomics, have become indispensable tools and provided valuable insights into the structure, composition, and functional dynamics of microbial communities. However, microbiota heterogeneity is largely ignored in these methods. The development of single‐cell approaches has advanced our understanding of the heterogeneity in microbial community function and structure.

### Single‐cell genomics

Single‐cell genome sequencing stands out as a groundbreaking milestone in culture‐independent approaches for microbial discovery and identification. Single‐cell genomics analysis investigates genomic heterogeneity by detecting the dynamic changes in DNA. This culture‐independent technique allows researchers to delve into the genetic material of individual microbial cells, enabling them to capture the genetic heterogeneity within the populations.

**Figure 2:**
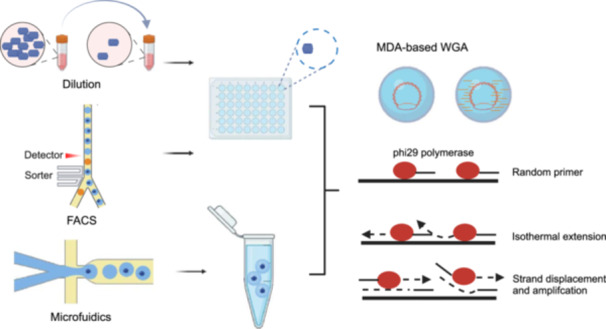
Profiling the heterogeneity of microbial populations by single‐cell genome sequencing. The schematic diagram illustrates the frequently used techniques utilized in the cell isolation process for single‐cell genome sequencing, including manual dilution, microdroplet emulsion formation, and fluorescence‐activated cell sorting (FACS). The diagram also provides procedural illustrations for the MDA‐based whole‐genome amplification (WGA) of the targeted cells.

Single‐cell isolation is the first step of single‐cell sequencing. Various technologies are available for isolating microbial cells in single‐cell genome sequencing, which can be broadly categorized into two classes: random encapsulation and micromanipulation. Manual dilution, microdroplet emulsion, and fluorescence‐activated cell sorting (FACS) are frequently used random encapsulation technologies (Figure 2). Micromanipulation techniques that rely on microscopic visualization, such as micro‐pipetting, microfluidic flow control, and laser capture microdissection, are widely used methods for single‐cell isolation. FACS is one of the most prevalent and high‐throughput techniques for isolating single cells and obtaining their genomes, even for environmental microbes that are difficult to cultivate^9^. Nevertheless, it does come with a limitation of the requirement of specialized and costly equipment. Moreover, miniaturizing the amplification reaction volume to the nanoliter range is also challenging. Microfluidics is an essential tool for single‐cell multi‐omics methodologies. The core components of microfluidics for single‐cell isolation often include droplet‐based systems, digital microfluidics (DMF), valves, and microwells, which serve as boundaries or spatial separators between individual cells. This technology facilitates lysis and other reactions necessary for DNA and RNA sequencing of the target cell within a visual and enclosed system. New methods with low cost and high throughput are developed for single‐cell isolation. For example, the virtual microfluidic approach developed by Xu et al. is cost‐effective because it does not require specialized equipment^10^. In this method, a dilute cell suspension is solidified within a poly (ethylene glycol) hydrogel, and the immobilized amplification products can be recovered using nanoliter‐range gel punches. In 2017, Lan et al. introduced an ultra‐high‐throughput approach based on droplet microfluidics^11^. Single cells are captured in individual hydrogel microspheres, and their genomes are then fragmented, barcoded, pooled, and sequenced together. Recently, flotation and interdigitated electrode forces on droplets to enable lasting system integrity (FIDELITY) was developed as a method to maintain system stability during droplet manipulation. A size‐selective droplet bandpass filtration mechanism is enabled by exploiting the inherent buoyancy of aqueous droplets and precisely focused dielectrophoretic forces from interdigitated electrode arrays, achieving high sensitivity and accuracy^12^. We anticipate that these emerging technologies in droplet microfluidic will have a significant impact on single‐cell profiling.

Another advantage of single‐cell genomics is its capability to capture and amplify all the genetic materials present within a single cell in the femtogram range. It creates a crucial connection between the genome of microbes and the plasmids and viruses contained within the same cell. This linkage enables a series of studies aimed at uncovering endo‐symbiotic associations, bacteriophage–host interactions, and cell–cell physical associations. The commonly used whole‐genome sequencing (WGS) technique, such as multiple displacement amplification (MDA), is based on isothermal amplification^13^, which utilizes the bacteriophage phi29 polymerase and random hexamer primers to amplify the DNA of a single cell (Figure 2). WGA‐X, taking advantage of a thermostable mutant phi29 polymerase, improves genomic recovery from single cells and viral particles with high GC content^14^. Almost all single‐cell microbes have been sequenced using isothermal amplification techniques. In 2005, Raghunathan et al. first sequenced the genome of a single *E. coli* cell and recovered ~30% of the genome from single, flow‐sorted bacterial cells by the MDA reaction^15^. 2 years later, Podar et al. and Marcy et al. applied this technology to *Saccharibacteria candidate* and showcased that single‐cell genome sequencing could be used for the identification of unculturable taxonomies^16^. Nowadays, researchers have successfully captured the genomes of numerous rare species using single‐cell sequencing. In 2022, Zheng et al. separated individual bacterial cells within droplets and applied MDA to obtain single amplified genomes (SAGs). They reconstructed the horizontal gene transfer (HGT) network and captured HGT events between 92 species pairs by sequencing over 20,000 microbial single cells^17^. Recently, a high‐efficiency phi29 DNA polymerase was introduced that lowers the bias of MDA and thus improves the sequence coverage of single‐cell genomics for microbiota samples^18^. These studies show that single‐cell genome sequencing could provide valuable insights into the mechanisms driving evolution and genetic heterogeneity within microbial communities. This groundbreaking approach has significantly advanced the functional prediction and phylogenetic identification of microorganisms, leading to a substantial expansion of the diversity represented on the phylogenetic tree of microorganisms.

Other single‐cell whole‐genome amplification (WGA) methods are also available for eukaryotes but have not been applied in bacteria in part due to the small genome size, high supercoiling, and species‐specific DNA methylation in bacteria. Degenerate oligonucleotide‐primed polymerase chain reaction (DOP‐PCR)^19^, a pure PCR‐based WGA method, uses degenerate primers to enable large‐scale genomic analysis. However, this approach has limited genome coverage completeness. While DOP‐PCR demonstrates high efficacy for detecting copy number variations (CNVs) across extensive genomic regions, more recent advancements utilize displacement pre‐amplification to produce PCR‐amplifiable templates. Representative techniques, including multiple annealing looping‐based amplification cycles (MALBACs)^20^ and PicoPLEX^21^, have been successfully demonstrated in mammalian single‐cell research. Linear amplification via transposon insertion (LIANTI) uses T4 RNA polymerase to linearly amplify the original template hundreds of times, and it was reported to have the highest coverage of a human diploid cell genome^22^. While these methods are promising, successful adaptation to microbial single‐cell studies will require optimization of cell lysis protocols to address the structural complexity of bacterial cell walls and minimize amplification bias to achieve high genome coverage and accuracy.

### Single‐cell transcriptomics

Various microbial community phenomena, including spore formation, quorum sensing, and conditional pathogenicity, have been shown to rely on cellular heterogeneity for their functionality. Single‐cell RNA sequencing (scRNA‐seq) has become a routine method for eukaryotic research in discovering new cell types or physiological states with specific gene expression, while scRNA‐seq technology for prokaryotic microorganisms showed limited breakthroughs until recently.

There are several technical challenges in bacterial single‐cell transcriptomics. First, the cell lysis procedure is limited by the fact that bacteria typically possess a thick bacterial envelope. In the context of a meta‐community, which consists of multiple species, including both Gram‐positive and Gram‐negative bacteria, as well as cells from different growth stages, the lysis procedure may lead to a low retention rate. This is due to differences in lysis efficiency, which can potentially introduce bias for certain cell states, such as the spore stage and the biofilm stage. Furthermore, the loss of certain species during the library preparation stage is another concern. Second, the RNA in single bacterial cells is approximately 100 times less than that in typical eukaryotic cells. While the detection limit of most eukaryotic scRNA‐seq approaches is 10 copies, the mean copy number of mRNAs in bacterial cells is significantly lower (0.4 copies per cell)^23^, ^24^. In addition, the half‐life of bacterial mRNA, which is in the minute range, is much shorter than that of eukaryotes. While constituting up to 98% of bacterial RNA, ribosomal RNA (rRNA) typically offers limited insights into cellular functional states^25^. Although targeted rRNA depletion protocols, including depletion of abundant sequences by hybridization (DASH), RNase H‐mediated cleavage and subtractive hybridization, and CRISPR‐based rRNA depletion, are available^26^, more efficient rRNA depletion methods are still required. Finally, there is no poly(A) tail on bacterial transcripts; therefore, they cannot be captured and amplified using oligo(dT) priming. Despite the above challenges, researchers have migrated the protocols developed for eukaryotic scRNA‐seq to bacterial studies.

**Figure 3:**
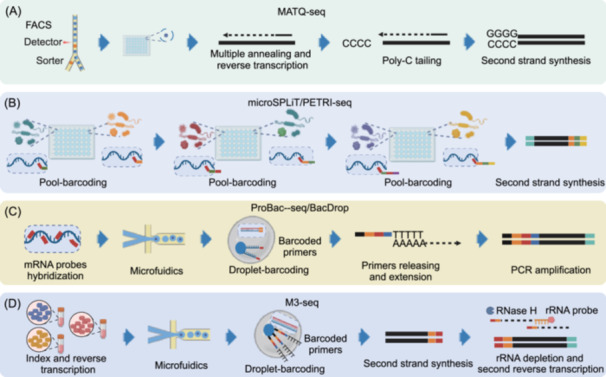
scRNA‐seq techniques of microbial populations. (A) Schematic of the multiple annealing and tailing‐based quantitative scRNA‐seq (MATQ‐seq) experimental workflow for bacterial cells. First, bacterial cells are sorted using FACS into a plate containing lysis buffer. Following cell lysis, the process is continued with multiple annealing with random primer and cycling reverse transcription, poly‐C tailing, second‐strand synthesis, and amplification. (B) Schematic diagram of sample preparation steps for split‐pool ligation‐based transcriptome sequencing (SPLiT‐seq), such as microSPLiT and prokaryotic expression profiling by tagging RNA in situ and sequencing (PETRI‐seq), which include three‐step pool‐barcoding labeling procedures, allowing for barcoding of the transcripts in single cells. (C) Schematic of droplet‐based approaches compatible with the 10× Genomics platform. Genome‐specific oligonucleotide probes with a 3′ poly‐A tail are used to target the mRNA within bacterial cells. The bacterial cells are then encapsulated into microfluidic droplets, where the mRNA‐probe hybridized complex from each cell is captured and barcoded, following the protocol of primers' release, extension, and PCR amplification. (D) Schematic of the M3‐seq experimental workflow. Transcripts are reverse‐transcribed in situ using indexed primers that carry unique sequences to label cells from different treatments. Cells are subsequently collected, mixed, and then dispensed into microfluidic droplets for a second round of indexing, which is achieved by ligating them with barcoded oligonucleotides. After RNA library amplification, ribosomal sequences are depleted by hybridizing them to complementary DNA probes, which are then cleaved by RNase H, reverse‐transcribing the remaining sequences, and ligated with sequencing adaptors.

For example, researchers used multiple annealing and tailing‐based quantitative scRNA‐seq (MATQ‐seq, Figure 3A)^27^, which was originally developed for eukaryotic scRNA‐seq, to profile the single‐cell transcriptome of *Salmonella typhimurium* and *Pseudomonas aeruginosa* ^28^. They used a highly sensitive random‐hexamer priming‐based scRNA‐seq protocol, captured about 200 transcripts for each individual cell, and profiled their growth‐dependent gene expression patterns.

Prokaryotic expression profiling by tagging RNA in situ and sequencing (PETRI‐seq, Figure 3B)^29^ is another successful approach developed based on the eukaryotic scRNA‐seq protocol named “SPLiT‐seq” for split‐pool ligation‐based transcriptome sequencing. By leveraging in situ combinatorial indexing methods to uniquely barcode individual bacterial cells, PETRI‐seq significantly increased the throughput of bacterial scRNA‐seq, enabling the profiling of tens of thousands of bacterial cells. Moreover, PETRI‐seq has demonstrated high purity and low bias in capturing the transcriptomes of both Gram‐negative and Gram‐positive bacteria. A recent refinement of PETRI‐seq, termed RiboD‐PETRI^30^, incorporates a ribosomal RNA‐derived cDNA depletion (RiboD) strategy, which effectively removes unwanted rRNA sequences from single‐stranded cDNA pools. This method is cost‐effective, does not require specialized instrumentation, and is compatible with high‐throughput workflows. By eliminating rRNA‐derived reads and improving mRNA detection rates up to 92%, RiboD‐PETRI enables more precise and sensitive profiling of bacterial gene expression at the single‐cell level. The application of RiboD‐PETRI has been demonstrated in studies of biofilm heterogeneity, where it enabled the identification of distinct subpopulations marked by unique genes.

Another method, microbial split‐pool ligation transcriptomics (microSPLiT, Figure 3B)^31^, is also adapted from ‘SPLiT‐seq’. Researchers performed in situ polyadenylation using *Escherichia coli* poly(A) polymerase I (PAP) to enrich the mRNA. To model Gram‐positive (*Bacillus subtilis*) and Gram‐negative (*E. coli*) bacteria, microSPLiT single‐cell sequencing detected median mRNA loads >300 transcripts per bacterium. They have captured rare sub‐populations by analyzing >25,000 individual cells, indicating that scRNA‐seq represents a useful and sensitive tool for profiling the heterogeneity of bacterial communities.

In 2023, two droplet‐based approaches that are compatible with the 10× Genomics platform, i.e., probe‐based bacterial sequencing (ProBac‐seq, Figure 3C)^32^ and bacterial droplet‐based single‐cell RNA‐seq (BacDrop)^33^, have been proposed. ProBac‐seq utilizes genome‐specific oligonucleotide probes with a 3′ poly‐A tail to target the mRNA in bacterial cells. The mRNA:probe hybridized complex of each cell is subsequently captured and barcoded, following a protocol similar to barcoding the transcriptome of eukaryotic cells using the 10× Chromium Controller. On the other hand, BacDrop uses a slightly different approach. It begins with one round of plate barcoding, followed by cDNA polyadenylation at the 3’ end. The cells are then loaded into droplets containing barcoded primers on 10× gel beads. Each droplet typically contains three to six single cells, and the transcript of each individual cell can be identified by the combination of plate barcodes and droplet barcodes. By using genome‐specific probes, ProBac‐seq can profile ~300 mRNA per cell without wasting sequencing on ribosomal or other non‐mRNA transcripts. However, the probe design limits the usage of ProBac‐seq to well‐known genome model organisms. While BacDrop can only capture less than 100 mRNA per cell, the increase in throughput by pre‐plate barcoding ensures sufficient recovery of rare cellular subpopulations or microbial species.

Another droplet‐based, high‐throughput single‐microbe RNA‐seq method introduced in 2023 is smRandom‐seq^34^, which offers a more universal, transcriptome‐wide profiling strategy without the need for genome‐specific probe design. This method achieves high species specificity, a low doublet rate, and sensitive gene detection, with a median of approximately 1000 genes detected per single *E. coli* cell. smRandom‐seq utilizes random primers for in situ cDNA synthesis, droplet microfluidics for single‐cell barcoding, and CRISPR‐based depletion for reducing rRNA content and enriching mRNA. Unlike probe‐based approaches, its genome‐independent design enables robust profiling across diverse microbial taxa. smRandom‐seq has also witnessed early commercial adoption and has been applied to uncover transcriptional heterogeneity in complex environments, including the rumen microbiome^35^, host–phage associations^36^, and keystone species^37^ in the human gut.

More recently, massively parallel, multiplexed, microbial sequencing (M3‐seq, Figure 3D)^38^ was developed, with post‐hoc rRNA depletion; this is a cell indexing scRNA‐seq platform for bacteria. M3‐seq enables the profiling of bacterial cells from different species under different treatments in a single sequencing run. Additionally, M3‐seq depletes rRNA after library amplification, with reduced risk of losing unamplified, non‐rRNA transcripts, and potentially increasing sequencing sensitivity. M3‐seq profiling of *B. subtilis* and *E. coli* uncovered rare subpopulations, delineated stress–response bet‐hedging mechanisms, and resolved phage infection dynamics.

scRNA‐seq has become an indispensable tool for investigating the heterogeneity of microbial communities. Although most of these approaches can capture around 200–300 mRNAs per individual cell, which represents approximately 5% of the total mRNA copies in a typical cell, they still enable us to identify rare sub‐populations and their marker genes by profiling tens of thousands of cells. Additionally, even with fewer mRNA molecules, analyzing a larger number of cells still allows us to uncover transcriptome heterogeneity within the microbial community.

### Spatial sequencing

In situ sequencing (ISS), originally described by Ke et al. in 2013^2^, enables the identification of mRNA molecules in tissues through the use of multiplexed padlock probes, a technique extensively applied in eukaryotic cells^39^, ^40^. In microbial community research, ISS is particularly valuable for exploring the interactions between hosts and microorganisms. As an illustration, researchers have utilized a combination of in situ capture techniques using spot sampling to obtain the host transcriptome, coupled with 16S rRNA sequencing to identify the taxonomic composition of the microbial community.

**Figure 4:**
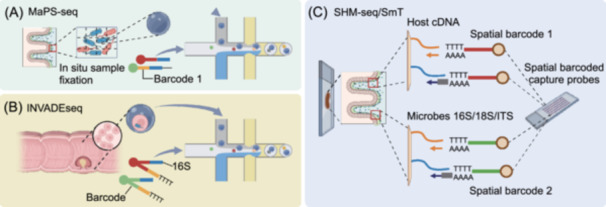
Sequencing‐based spatial profiling of microbial communities at the single‐cell level. (A) Schematic of the MaPS‐seq experimental workflow. The spatial metagenomic analysis by plot sampling and sequencing intestine embedded in a gel matrix and cryofracturing them into particles are illustrated. Neighboring microbial taxa in the particles are then identified through droplet‐based encapsulation, where the microbes 16S are captured by barcoded beads. Then, the following is the protocol of PCR amplification and sequencing. (B) Schematic of the INVADEseq experimental workflow. Tumor samples are processed using the gentle magnetic activated cell sorting (MACS)‐quality Octo Dissociator to obtain single‐cell suspensions, and then the cell suspension is processed with the 10× Chromium controller to capture single cells within a gel bead emulsion containing a master mix with two primers: one targeting the polyadenylated host mRNA and the second targeting the bacterial 16S rRNA gene. Following reverse transcription (RT), the libraries are prepared and sequenced. (C) Schematic of the SHM‐seq/SmT experimental workflow. Tissue sections from mouse colons are placed on a barcoded glass array, with a barcoded surface adapted for capturing both polyadenylated host mRNA and 16S/18S bacterial rRNA, ITS fungal rRNA. Tissue sections are imaged, cells are permeabilized, and cDNA is synthesized on the array surface before library preparation and sequencing.

In 2019, a culture‐independent method named metagenomic plot sampling by sequencing (MaPS‐seq)^6^, was developed to characterize the microbiome spatial organization at a micrometer‐scale resolution (Figure 4A). This method involves immobilizing intact microbiome samples in a gel matrix and cryofracturing them into particles. Neighboring microbes in the particles are then profiled through droplet‐based encapsulation, barcoded 16S rRNA amplification, and sequencing. By analyzing various mouse intestine regions, the heterogeneity in microbial community distributions was displayed with bidirectional taxon–taxon interactions. Universal associations involving *Bacteroidetes* were identified throughout intestinal compartments, and dietary interventions were shown to be associated with localized phylogenetic clustering. This study demonstrates the potential of spatial metagenomics in studying microbial biogeography in complex habitats, providing a powerful tool for understanding the spatial organization of microbiomes and the interactions that drive their functions.

In 2022, a novel in situ spatial‐profiling and scRNA‐seq method called invasion–adhesion‐directed expression sequencing (INVADEseq)^41^ was also developed to identify host‐associated bacteria and the host cell transcriptomics (Figure 4B). Using 10× Visium spatial transcriptomics, intratumoral microbiota composition and niche localization in patient‐derived tissue specimens were identified. Applied to neoplastic tissues, this approach mapped bacterium–host cell interactions while concurrently identifying dysregulation in key pathways governing inflammation, metastasis initiation, cellular quiescence, and DNA repair fidelity. GeoMx digital spatial profiling further revealed bacterial colonization of immunosuppressive hypovascular niches. Crucially, these microbially colonized regions showed lower Ki‐67 expression in malignant cells relative to microbiota‐depleted tumor areas. This study provides valuable insights into the complex interactions between the host and its microbiota within the tumor microenvironment and offers potential new targets for therapeutic intervention.

Recently, a method called spatial host–microbiome sequencing (SHM‐seq)^5^ was developed to explore the host–microbe interaction (Figure 4C). This spatially resolved sequencing platform integrates tissue histology, poly(A)^+^ RNA capture, and bacterial 16S profiling via engineered barcoded substrates. Applied to murine gut, deep learning‐based spatial mapping revealed micro‐niches defined by host–microbiota topological coordination. Subpopulation‐specific gene expression patterns in gut cells, shaped by distinct micro‐environments and interactions with regional commensal bacteria, are key determinants of host–microbe interactions. SHM‐seq could be used to enhance the study of host–microbe interactions in health and disease, particularly in gut mucosal and barrier tissues, where microbes closely interact with the host. These interactions prevent pathogen colonization and support symbiosis.

A novel approach to explore the intricate spatial interactions between hosts and related microbes was reported recently. The authors presented spatial metatranscriptomics (SmT)^42^, a sequencing‐based technique that utilizes 16S/18S/ITS/poly‐d(T) multi‐modal arrays to simultaneously characterize both the host transcriptome and the microbiome in tissues at a 55‐micrometer resolution (Figure 4C). They demonstrated the application of SmT in outdoor‐grown *Arabidopsis thaliana* leaves. The authors identified tissue‐scale bacterial and fungal hotspots and utilized network analysis to investigate inter‐ and intra‐kingdom spatial interactions among microorganisms, and also the host's response to these microbial hotspots. The development of SmT represents a promising tool to obtain answers to fundamental questions about the interplay between host and their microbes, potentially enhancing our understanding of host–microbe interactions within plant or animal tissues.

### LCM‐coupling sequencing

LCM is a powerful tool to isolate and study the heterogeneity in cells or tissues. Coupled with NGS, microarray analysis, or qRT‐PCR, this approach deciphers compartment‐specific gene expression patterns. For instance, researchers have used LCM‐RNA‐seq to systematically identify comprehensive landmark gene sets without prior selection bias. This approach serves as a scalable alternative to FISH‐based single‐cell spatial reconstruction^8^. However, it has not yet been adapted for microbiota studies.

## MOLECULAR IMAGING‐BASED SINGLE‐CELL PROFILING TECHNIQUES

In recent years, molecular imaging, especially spectral imaging, has garnered significant attention and found widespread application in cell heterogeneity analysis for eukaryotic cells. This technology seamlessly integrates spectral technology, image processing technology, and computer pattern recognition technology, enabling accurate analysis of cellular structure, function, and metabolic state. Commonly used spectral techniques include fluorescence spectroscopy, infrared spectroscopy, Raman spectroscopy, and mass spectrometry (MS). These spectral techniques can analyze biochemical molecules, proteins, nucleic acids, and other components within cells, providing more detailed insights into cellular research. Cell heterogeneity analysis techniques based on spectral imaging offer high resolution, high sensitivity, and high accuracy, effectively analyzing cellular stress responses, metabolic activity, growth characteristics, etc.

### Spectral encoding FISH

Various microscopy techniques can visualize the morphological characteristics of cells. By using fluorescent dyes such as DAPI and SYBR GREEN, researchers can identify and map the distribution of microbes within environmental and host‐associated samples. However, the lack of specificity limits their application in microbial community studies. Currently, FISH is extensively used for the detection and identification of microbes across diverse microbiology fields. FISH is designed to target the 16S rRNA of bacteria, enabling the visualization and localization of microbes in various environments. Several strategies have been developed to enhance the multiplexing capabilities of FISH, allowing for the identification and spatial mapping of multiple microbial taxa within a microbial community. These strategies provide insights into the spatial organization and interactions of microbial species.

By using fluorescent‐labeled oligonucleotide probes that are targeted to the rRNA of microbes, researchers can selectively label specific taxons and further accurately quantify their cell numbers. In general, the oligo‐probe needs to be designed with appropriate length, GC content, and specificity. A variety of software tools can be used for designing oligonucleotide probes targeted to the desired microbial taxa of interest, such as ARB Probe Design^43^ and DECIPHER^44^. In addition, tools such as probeCheck^45^ or mathFISH^46^ can be used to test the specificity and binding efficiency of the probe. Various oligonucleotide probes have been designed for general usage such as probes targeted to the conserved region of 16S rRNA of bacteria. The probes that have been reported to be used in the literature can be found in probeBase^47^.

FISH has been widely used to label individual microbial cells across various samples. In 2020, the Staussman research group utilized a universal probe targeted to bacterial 16S rRNA to detect endophytic bacteria within tumor cells^48^. By utilizing specific probes designed for different microorganisms of interest, FISH enables the study of spatial structures within complex microbial communities. However, a key limitation of this method arises from the spectral overlap of multiple fluorophores, restricting its applicability to small synthetic communities or studies focusing on a selection of few key species within the community. Two main strategies have been developed to extend the number of target species that can be marked simultaneously, that is, combinational labeling and sequential labeling.

**Figure 5:**
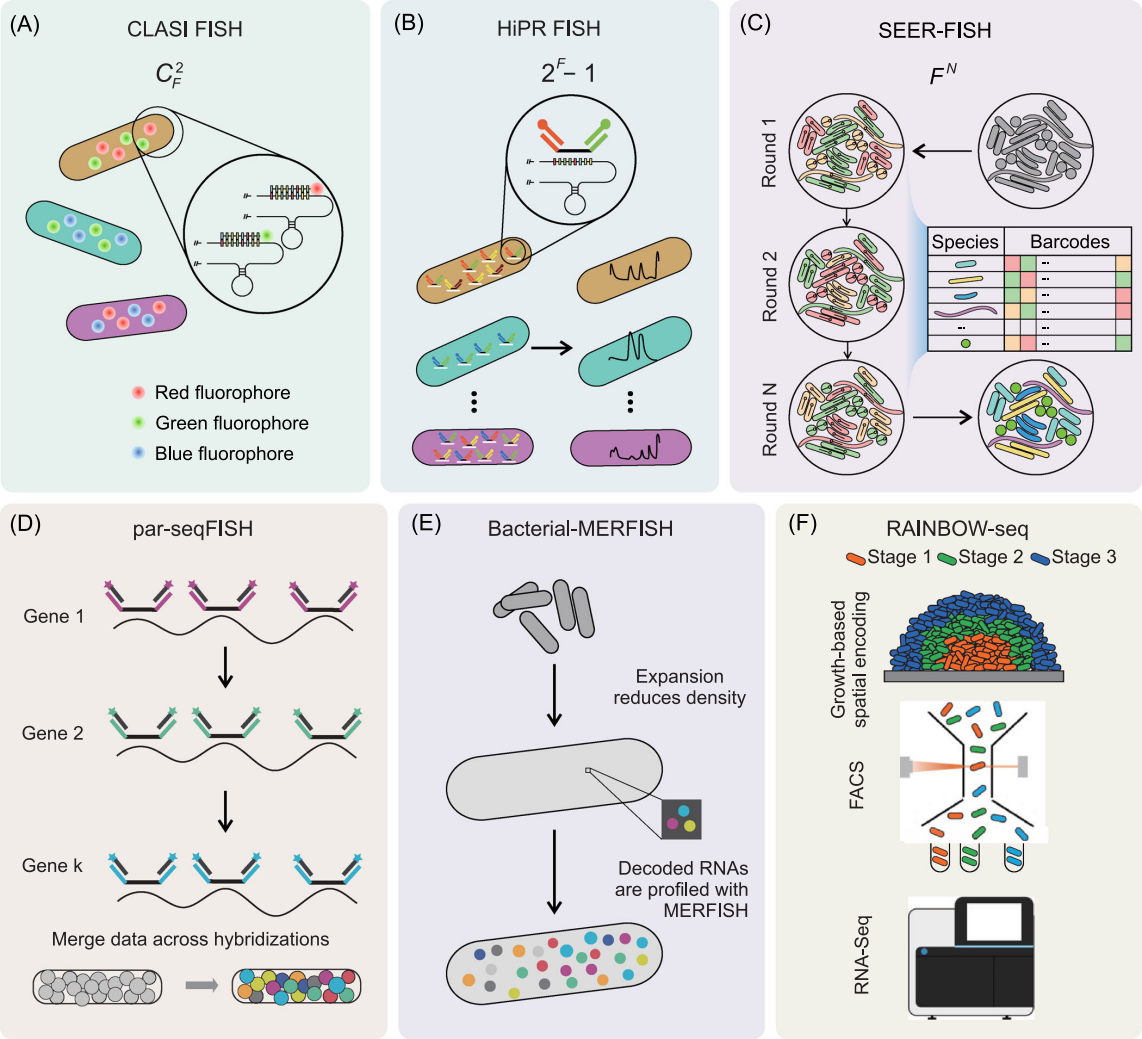
Image‐based spatial profiling of microbial populations and communities at the single‐cell level. (A) Design of CLASI‐FISH. Each bacterial is hybridized with two species‐specific probes labeled with fluorophores. Through combinatorial labeling with *F* different fluorophores, up to $C_{F}^{2}$ different species can be distinguished. Image adapted from reference^50^. (B) Design of HiPR‐FISH. Each bacterial species is targeted using species‐specific probes containing unique readout sequences. Fluorescently labeled readout probes hybridize to the primary probes, generating a distinct spectral barcode for each species. Using *F* fluorophores enables the identification of up to $2^{F}-1$ species. Image adapted from reference^51^. (C) Design of SEER‐FISH. Each bacterial taxon is encoded with an *F*‐color, *N*‐bit barcode. Spatial distributions are revealed through *N* sequential rounds of FISH, with each round comprising probe hybridization, imaging, and dissociation. This allows detection of up to $F^{N}$ different species. Image adapted from reference^52^. (D) Design of par‐seqFISH. Target mRNAs are first hybridized with a set of primary, nonfluorescent probes. Gene expression is then measured using sequential hybridization with readout probes labeled with different fluorophores, enabling multiplexed spatial transcriptome profiling at the single‐cell level. Image adapted from reference^53^. (E) Design of Bacteria‐MERFISH. Bacterial cells undergo ~1000‐fold volumetric expansion to overcome high mRNA density. Subsequently, RNAs are profiled using MERFISH. Image adapted from reference^54^. (F) Design of RAINBOW‐seq. Growth‐dependent dyes are sequentially added to label cells within a biofilm based on growth dynamics. Cells are sorted using FACS based on their fluorescence. Transcriptomes are then profiled via RNA sequencing for each spatially resolved region. Image adapted from reference^55^.

Valm and colleagues introduced an innovative technique named combined labeling and spectra identification FISH (CLASI FISH)^49^, ^50^. By labeling one target by a combination of two fluorophores out of a set of eight possibilities, they achieved simultaneous labeling of $C_{8}^{2}=28$ targeted species (Figure 5A). Furthermore, the number of targeted species increased to 120 by expanding the same approach to include a set of 16 different fluorophores and using an improved spectral image analysis algorithm. In 2020, Shi et al. proposed high‐phylogenetic‐resolution microbiome mapping by fluorescence in situ hybridization (HiPR‐FISH)^51^, which utilized all possible combinations of 10 fluorophores and enabled identification of $2^{10}-1=1023$ isolates (Figure 5B). They used a two‐step hybridization scheme and a spectral unmixing algorithm based on machine learning and enabled characterization of microbial biogeography with high complexity and diversity.

However, the multiplicity of the approaches based on combinational labeling is still limited by the number of fluorophores, that is, $2^{F}-1$, where *F* is the number of fluorophores. In contrast, technology that sequentially labels targets according to corresponding barcodes allows for a much higher level of multiplicity, reaching $F^{N}$, where *N* is the length of barcodes. Sequential FISH was initially used to profile mRNAs in mammalian cells and has since demonstrated great success in spatial transcriptomic studies. Recently, Cao and her colleagues applied sequential FISH together with error‐robust barcoding schemes (Sequential error‐robust FISH, SEER‐FISH)^52^ to map the spatial structure of microbial communities (Figure 5C). The theoretical complexity of SEER‐FISH is remarkable, as demonstrated by up to 26 hybridization–dissociation cycles. They successfully mapped the biogeography of a microbial community consisting of 130 strains (90 taxa) on the *Arabidopsis rhizoplane*. Through this study, they demonstrated the clustering and associations of bacteria in response to rhizosphere exudate treatment.

Spatial heterogeneity is not only observed in microbial communities composed of multi species but also in genotype isotropic microcolonies. The phenotypic differences among individuals within a population enable collective resistance to external pressures and the maintenance of a growth advantage for the community through division of labor. The heterogeneous phenotypes, such as motility, growth, and survival rate, can be directly measured through microscopic inspection or in conjunction with fluorescent dyes and fluorescent proteins as reporters for marker genes. Recently, studies of heterogeneous gene expression have been revolutionized by the development of single‐molecule fluorescence in situ hybridization (smFISH)^56^. By utilizing FISH probes specifically designed to target mRNA molecules, it is now possible to accurately quantify mRNA at the single‐cell level, providing valuable insights into gene expression patterns and cellular heterogeneity.

In 2021, a significant advancement was made with the development of parallel sequential fluorescence in situ hybridization (par‐seqFISH)^53^, with spectrally distinct fluorophores in multi‐round hybridization–imaging–stripping cycles (Figure 5D), enabling concurrent microbial taxonomy resolution and single‐cell transcriptional profiling. Using par‐seqFISH, researchers captured the expression profile of 105 marker genes in *Pseudomonas aeruginosa* at a single‐cell resolution. More than 600,000 individual cells across various growth conditions, such as planktonic cells at different growth stages and cells within microcolonies during biofilm formation, were analyzed. The development of par‐seqFISH provides new possibilities for studying the spatial and functional heterogeneity of microorganisms in various ecological niches and facilitates the exploration of gene‐regulatory networks and the identification of novel cellular states within microbial communities.

Recent advancements have integrated expansion microscopy with multiplexed error‐robust FISH (MERFISH), resulting in the development of bacterial‐MERFISH^54^ (Figure 5E). This technique enables up to ~1000‐fold volumetric expansion of bacterial cells, effectively addressing the high intracellular RNA density that traditionally hampers spatial transcriptomic analysis in prokaryotes. Researchers have applied bacterial‐MERFISH to investigate *E. coli*'s transcriptional response to a shift in the carbon source from glucose to xylose. The analysis revealed a highly heterogeneous response among individual cells, where *E. coli* dynamically explored multiple carbon‐utilization operons before committing to those associated with xylose metabolism. Furthermore, the subcellular resolution of bacterial‐MERFISH enabled detailed mapping of the intracellular spatial organization of the *E. coli* transcriptome, uncovering diverse spatial patterns influenced by genomic and proteomic architecture. Bacterial‐MERFISH was also used to study the spatial gene expression dynamics of the human gut commensal *Bacteroides thetaiotaomicron* in the mouse colon.

In summary, FISH‐based approaches offer unique advantages in profiling the microbial communities at a single‐cell resolution, particularly in the investigation of spatial heterogeneity. In parallel, sequencing‐based methods such as RAINBOW‐seq^55^. (Figure 5F) complement FISH approaches by enabling spatially resolved transcriptome profiling through growth‐based labeling, FACS, and RNA sequencing, thereby linking cellular states to spatial context with high molecular resolution. By combining and improving these techniques, such as rRNA FISH probe design with high specificity and the ability to target various genetic levels^44^, as well as different error‐robust coding schemes for both rRNA and mRNA targets with increased multiplexity and throughput, these approaches can further provide valuable insights into the organization, interactions, and functional characteristics of individual cells within complex biological systems.

### Metabolic labeling‐based imaging

Metabolic labeling enables selective labeling of specific bacterial populations or structures, such as cell walls or membranes, using chemically tagged unnatural precursors or substrate mimics, as well as stable isotopes. This approach, when coupled with click chemistry to introduce fluorescent dye, allows for the specific labeling of bacteria, subgroups within the bacteria, or the entire commensal microbiota. This enables the tracking of their metabolic activity, colonization, and dissemination. By selectively labeling, researchers can investigate the functions and regulation of specific components within bacteria, facilitating the study of bacterial physiology and identification of potential drug targets. This method also enables isolation and analysis of targeted bacterial populations, providing valuable insights into their biological processes.

As an example, d‐amino acid metabolic labeling was developed to label the peptidoglycan layer of bacterial cell walls. Using sequential tagging with fluorescent d‐amino acid metabolic probes (STAMP)^57^, Wang et al. established a novel tracking system to monitor viability dynamics of transplanted microbiota during fecal microbiota transplantation (FMT). Based on the method, they separated and identified the surviving transplanted bacteria by FACS and 16S sequencing, and then evaluated the effects on transplant survival by different antibiotics pretreatments on recipients. Integration of STAMP with FISH staining enabled in vivo visualization of growth and division dynamics across murine gut microbiota at multiple taxonomic ranks, including uncultivatable species refractory to in vitro study^58^. Additionally, by combining the tissue‐clearing technique with metabolic labeling, Wang et al. achieved quantitative 3D imaging with single‐cell resolution of both indigenous microbiota and transplanted bacteria within their native context of intestinal architecture^59^.

In addition to d‐amino acid labeling, metabolic oligosaccharide engineering is another technique used for bacterial labeling. Kasper's group^60^ applied this method, along with bio‐orthogonal click chemistry, to label bacterial polysaccharides of various commensal microbes, including *B. fragilis*, an immunologically important commensal bacterium. Using this approach, they were able to detect and differentiate the distribution and colonization preferences of three *Bacteroides* species tagged by spectrally distinct fluorophores throughout the intestine. This technique also permits the study of host–microbe interactions such as characterization of the interactions of intact microbes and their immunogenic polysaccharides within myeloid and B lymphocyte populations.

Moreover, bacteria can be selectively targeted by other responsive metabolites. Johnson's group demonstrated this by treating mice with bio‐orthogonal labeling cholesterol, which enabled the identification and isolation of diet‐derived cholesterol‐engaging microbes using FACS^61^. Their work sheds light on *Bacteroides*’ functional involvement in host cholesterol‐3‐sulfate metabolism, providing critical insights for diet–microbiome interaction studies across physiological systems.

Expansion of the repertoire of available probes to cover a wider array of bacterial metabolic functions—including nucleic acid metabolism, lipid biosynthesis^62^, energy production, and signaling pathways—is an active area of research. Development of more pathway‐specific and sensitive metabolic probes will not only deepen our understanding of bacterial physiology in complex communities but also facilitate identification of novel therapeutic targets. Future advances in probe design, coupled with improvements in bio‐orthogonal chemistry and imaging techniques, have the potential to broaden the applicability of metabolic labeling and enable more comprehensive, dynamic mapping of bacterial metabolism at a single‐cell resolution.

### Genetic labeling‐based imaging

Based on synthetic biology techniques, genetic labeling with reporter genes mostly expressing fluorescent protein or luciferase is widely used in bacterial imaging techniques. However, this approach has limitations, as it requires the species to be culturable and genetically easy to manipulate, and stable colonization of the strain is required for localization studies of bacteria in vivo. Therefore, genetic engineering strategies are currently limited to use in only a very small proportion of bacteria.

Sonnenburg's group recently developed a synthetic biology platform enabling combinatorial expression of orthogonal fluorescent proteins in high‐abundance *Bacteroides* in the gut^63^. This enabled the simultaneous imaging of six different *Bacteroides* strains in the mouse gut. Comparative analysis of simultaneous versus sequential bacterial colonization revealed that pioneer advantage governs intestinal niche occupancy capacity, demonstrating crypt‐specific exclusion of secondary colonizers. This study deciphers strain‐level spatial heterogeneity within gut microbial ecosystems.

### Single‐cell Raman spectroscopy (SCRS) for phenotype sorting and analysis

Raman spectroscopy technique allows for simultaneous, noninvasive, and label‐free detection of different metabolic products. Each single‐cell Raman spectrum comprises over 1500 Raman peaks corresponding to different types of chemical bonds and these peaks can serve as a unique “molecular fingerprint” for each individual cell^64^. Single‐cell Raman spectroscopy (SCRS)^65^, ^66^ technology captures multidimensional biochemical fingerprints of intracellular constituents, establishing it as a robust label‐free phenotyping platform for individual cells.

A collection of single‐cell Raman spectra from a population or consortium is defined as Ramanome^64^, which can determine and measure the state and function of the system at a single‐cell resolution, as well as phenotypic heterogeneity^67^. At a single‐cell resolution, researchers have demonstrated that Ramanome can quantitatively detect the metabolic rates of cells using substrates such as hydrogen^68^ and carbon^69^. Also, the diversity and content of various Raman‐sensitive products such as pigments, triglycerides, starch, and proteins can also be determined^70^. By integrating with Raman‐activated cell sorting (RACS) platforms, sorting of specific phenotype cells has been realized including carotenoid‐producing bacterial cells^71^, astaxanthin (AXT)‐hyperproducing microalgae cells^72^, and triacylglycerol (TAG) ‐producing yeast cells^73^. Based on a reference SCRS database of 21 statutory probiotic species, Xu et al. achieved species‐level identification with 93% accuracy for each cell directly extracted from probiotics products^74^. The subsequent single‐cell Raman‐activated cell sorting (scRACS) and Sequencing produce indexed genome assemblies precisely based on one cell and can reach ~99.40% genome‐wide coverage. Building on this, fluorescence in situ hybridization‐guided scRACS‐seq (FISH‐scRACS‐seq), a phylogeny metabolism dual‐directed single‐cell genomics approach, was recently developed to bridge the gap between microbial markers and their in situ functional mechanisms^75^. Moreover, in clinical gastric biopsy samples at precisely single‐cell resolution, Xu et al. accomplished rapid pathogen identification, antimicrobial susceptibility test, and high‐quality WGS of cells with levofloxacin or clarithromycin resistance phenotypes^76^, ^77^. In summary, the Ramanome is a versatile technique that can detect and quantify products, measure substrate and metabolite profiles, discriminate between cell types or states, and characterize stress responses and model environmental changes at a single‐cell resolution. Additionally, by coupling metabolic profiling with downstream genome sequencing (scRACS‐Seq^71^, ^76^, ^78^, ^79^) or culture (scRACS‐Culture^80^), it bridges phenotypes and genotypes at the ultimate resolution of one cell, a paradigm advanced decisively by FISH‐scRACS‐seq^75^. to dissect microbiota functions with ecological relevance.

However, a Raman peak in the Raman spectrum usually refers to a class of substances with the same or similar chemical bonds, while it is difficult to distinguish specific proteins, nucleic acids, fats, pigments, and so forth^65^. Although SCRS provides a panoramic phenotype, phenome analysis needs to be demonstrated in relation to specific applications. Application of deep learning algorithms in Raman analysis provides a novel way to distinguish different substances within the same class. These algorithms have been widely used to distinguish different phenotypes, which also presents a challenge in constructing data algorithms that are based on the full spectrum of Raman^81^, ^82^, ^83^, ^84^.

### Infrared spectroscopy‐based single‐cell/spatially explicit methods

Optical photothermal infrared (O‐PTIR) spectroscopy enables single‐bacterium biochemical profiling by acquiring infrared spectral fingerprints, resolving molecular‐level data on microbial metabolites, pathway intermediates, and metabolic end‐products^85^, ^86^. This label‐free methodology circumvents the limitations of fluorescence‐based techniques, eliminating dependency on commercial probes prone to photobleaching and potential metabolic interference. In 2023, researchers introduced an application of O‐PTIR spectroscopy, which was specifically designed to investigate phenotypic heterogeneity among *Bacillus* strains by precisely measuring its infrared spectrum^87^. This study demonstrated the capability of O‐PTIR's to swiftly, in a label‐free manner, and semi‐quantitatively assess polyhydroxybutyrate (PHB) production within individual cells. This technique represents a significant step forward in understanding and analyzing microbial metabolism at a granular level.

Using an infrared micro‐spectroscopy‐based method, another research group revealed phenotypic divergence during biofilm development and interstrain heterogeneity in *E. coli* ^88^. Coupled with two‐dimensional correlation spectroscopy, this approach delineated sequential biomolecular transitions—notably primary polysaccharide restructuring—during biofilm maturation. This methodological advancement establishes label‐free infrared platforms for biofilm phenomics while enabling drug screening to modulate biofilm architecture and ecological dynamics.

### Microbial single‐cell MS

MS is a label‐free, versatile analytical method that enables the identification and quantification of a wide range of analytes, including proteins, and intra‐ and extracellular metabolites, which are usually not accessible by other spectroscopic methods. MS‐based metabolomics is one of the pivotal technologies for detecting and characterizing the microbial‐derived small molecule, providing insights into the functional roles of these microbial metabolites. Recent advances in MS, coupled with other technologies, such as microfluidic chips, have made it possible to assess the molecular heterogeneity of individual cells. By miniaturizing cell handling, incubation, and enhancing chip‐coupling technology with MS, MS approaches are now capable of analyzing microbes at a single‐cell level^89^. However, the term “single‐cell analysis,” particularly in the microbial context, often refers to the examination of numerous cells at microscopic scales, rather than a single, isolated cell. The minute quantities and high variability of target analytes present major challenges for the implementation of MS in true single‐cell analysis.

### In vivo single‐cell level dynamics in gut microbiota profiling

Advances in imaging modalities, molecular probes, and synthetic biology now enable real‐time in vivo monitoring of gut microbiota dynamics in living models. Bioluminescence imaging (utilizing endogenous ATP or exogenous substrates), near‐infrared fluorescence metabolic labeling, and photoacoustic tomography (PAI) collectively capture the spatiotemporal, genetic, and metabolic activity of microbial communities. For instance, luciferase‐engineered *E. coli* can be tracked via ATP‐dependent bioluminescence in murine intestines^90^, while near‐infrared (NIR)‐dye‐conjugated metabolites permit noninvasive microbiota tracing^91^. PAI further expands this capacity through genetically encoded reporters for volumetric molecular imaging^92^.

## CONCLUSION AND PERSPECTIVE

Here, we provide an overview of advanced approaches for profiling individual cell properties within microbial communities to address key research questions in microbiology. These innovative methods not only provide valuable insights into the heterogeneity of microbial communities but also highlight future directions and opportunities in the field.

Microbial heterogeneity has long been recognized and is evident in a wide range of phenotypes, such as varying degrees of virulence and antibiotic resistance. These heterogeneous traits are fundamental to species fitness and evolution, underpinning well‐known potential strategies such as bet hedging and division of labor. Microbial populations benefit from heterogeneity by becoming better equipped to survive in fluctuating environments and enabling functional specialization to exploit new ecological niches. Heterogeneity arises not only from genotypic changes, such as genome rearrangements and mutations, but also from epigenetic modifications, physiological states of the cell (e.g., cell‐cycle stage and age), and stochastic variation in gene expression. Recent developments in single‐cell technologies have begun to characterize the heterogeneity within human gut microbiota. These tools enable the identification and characterization of low‐abundant members, exploration of the intrinsic genomic and functional variability of microbes at the strain level, assessment of their responses to perturbations, examination of the spatial functions involved in community assembling, inter‐microbe interaction^93^, microbe–phage interaction, and host–microbe interactions involved in health and disease, thereby further enhancing our understanding of the human microbiome.

The gut microbiota shows longitudinal stratification along the gastrointestinal tract, driven by chemical gradients, nutrient availability, antimicrobial peptide distributions, and mucosal topography. Mucosal and luminal niches harbor phylogenetically distinct communities, while microscale patchiness reveals complex spatial architectures. Microbial social dynamics—including competitive interactions—operate across spatial hierarchies, from micron‐scale proximities to millimeter‐range signaling. Spatial heterogeneity in these interactions fundamentally shapes microbial population assembly and successional patterning^94^. Surveying the spatial heterogeneity of the gut microbiota, spatial distribution, and interactions among symbiotic microbes has the potential to enable understanding of the development and maintenance of a resilient gut microbial ecosystem. This, in turn, can provide insights into microbial spatial organization and support the rational design of synthetic ecosystems.

Technically, single‐cell microbiology relies on single‐cell isolation and manipulation. Microfluidic droplets for single‐cell isolation and library preparation are the most promising methods to profile the heterogeneity of microbial community at high throughput. These droplets, which enclose picoliter‐to‐microliter‐sized reaction chambers, enable the simultaneous measurement of DNA/RNA in thousands of cells with higher reaction efficiency, detection precision, and reduced costs due to minimized reagent usage. However, the challenges associated with the microfluidic droplet method include high cell loss, rendering it unsuitable for rare cell samples, and the inability to introduce multiple reagents, which limits its use in multistep procedures. Another widely used method is split barcoding, such as SPLiT‐seq and PETRI‐seq. In these techniques, cells are randomly distributed, and diverse barcodes are introduced in several rounds to ensure a unique barcode combination for each cell. The advantage of split barcoding techniques is that it does not require droplet‐based equipment and offers high throughput at low cost, with relative simplicity for laboratory replication. However, split barcoding also has some technical caveats, such as the need for careful experimental handling to ensure adequate cell separation and avoid cell doublets. Additional rounds of splitting and pooling also increase the risk of cell loss and aberrant ligation errors. There are also shared challenges for those methods, such as the lack of both universally compatible bacterial cell lysis techniques and robust reverse transcription (RT) protocols that do not depend on transcript polyadenylation.

Single‐cell genome sequencing can capture the extent of genomic strain‐level diversity within a community, which is difficult for metagenomic sequencing to achieve. Therefore, it can dissect the heterogeneity in natural populations and enable evolutionary analysis of the community^95^. For example, it can be applied to survey the horizontal gene transfer and phage–host interaction^17^, ^96^. Moreover, single‐cell genome sequencing is also capable of capturing low‐abundance species or non‐culturable species, facilitating the assembly of their genomes.

For scRNA‐seq, both reverse transcription and PCR amplification would cause bias, resulting in the distortion of the relative abundance of transcripts. Additionally, it has the capability to dissect heterogeneous populations of cells; for instance, it may permit transcriptomic profiling of rare cell subsets even with limited input material. Regarding the high prevalence of rRNA in microbes, whole‐transcript sequencing often results in a limited proportion of informative mRNA reads, even though rRNA‐targeted depletion protocols are currently implemented in some bacterial scRNA‐seq procedures. For the FISH‐dependent methods, such as par‐seqFISH, rRNA depletion is not required, but these approaches are limited by probe multiplexing constraints, and designing mRNA probes requires prior knowledge, which may be limited by the gene identified through bulk RNA‐seq, but not novel genes.

While basic single‐cell sequencing does not maintain spatial information of the cells, image‐based approaches, such as FISH, enable precise localization of the target molecules (DNA or RNA) under the microscope and have been applied to tackle this problem. However, FISH is usually limited by low throughput due to spectral overlap among fluorophores. To improve the multiplexing capacity for imaging gut microbiota, emerging encoding strategies have been proposed. Yet, increasing the number of labeling cycles also increases the encoding error rate and processing complexity. Well‐established smFISH approaches (e.g., par‐seqFISH) offer high detection efficiency but require longer RNA species and produce lower signal intensity compared to enzymatic amplification methods. These approaches are also limited in terms of coverage of the whole microbial transcriptome. Other pioneering in situ sequencing methods (e.g., MaPs‐seq and SHM‐seq) are currently restricted to detecting host spatial transcriptomics together with microbe taxonomy. Development of similar methods to further profile the transcriptome of both the gut microbiota and the host remains challenging but promising.

To enable functional insights into microbial subpopulations, integration of multiple single‐cell approaches offers a path forward. By combining single‐cell sequencing with techniques like FISH and Raman spectroscopy, the precision and throughput of single‐cell capture can be enhanced. For instance, specific subpopulations can be labeled through FISH or metabolic labeling, which facilitates enrichment of defined relevant taxa or subpopulations with functions of interest^97^, ^98^. Combining taxonomy‐based fluorescent labeling and FACS with single‐cell sequencing represents another strategy for improving the precision^99^. Recent advancements in spatial multi‐omics technologies, such as multi‐omics in situ pairwise sequencing (MiP‐seq)^100^, further expand our ability to characterize heterogeneity. MiP‐seq enables simultaneous detection of DNA, RNA, proteins, and other biomolecules at a subcellular resolution. It can be further integrated with in vivo calcium imaging and Raman imaging, supporting multidimensional analyses of molecular and functional maps. A particularly promising direction is the integration of metabolic labeling with Raman micro‐spectroscopy and single‐cell sequencing. This combination has the potential to directly link metabolic function to genetic identity in individual cells within complex environments, thereby bridging phenotype and genotype at a single‐cell resolution. These combined strategies enable a more detailed and accurate analysis of the transcriptional activities, metabolic states, and genomic characteristics of target cells within complex microbial communities. Despite technical challenges such as sensitivity limits and signal interpretation, such integrative approaches hold great promise for uncovering keystone functional species in complex microbial communities associated with chronic diseases or diverse environmental environments.

The cross‐sectional nature of conventional omics approaches (e.g., scRNA‐seq) inherently limits the detection of longitudinal community behavior, rendering temporal processes inaccessible. To overcome this hurdle, metabolic labeling and live imaging techniques have emerged as powerful tools to resolve spatiotemporal dynamics in microbial communities, overcoming traditional analytical limitations. Metabolic labeling allows for the tracking of the cellular processes to gain fundamental insights into cellular interactions such as cross‐feeding, infection, colonization resistance, and phage–bacteria interactions. Live imaging, on the other hand, which also provides real‐time visualization of cellular activities and behaviors of the microbial community, was already in use to evaluate the prebiotic colonization in the gut. Single‐cell stable isotope probing utilizes techniques such as Raman microspectroscopy or nanoscale secondary ion mass spectrometry (NanoSIMS) to achieve spatially resolved tracking of isotopic tracers across cellular substructures, molecular components, and metabolic pathways^101^, ^102^.

Advances in single‐cell characterization technologies have generated a growing volume of single‐cell data in microbial research, underscoring the urgent need for specialized analytical tools. For example, tools that integrate single‐cell transcriptomic data with spatial transcriptomics^103^ are essential for uncovering spatially resolved functional insights. Although numerous computational methods have been developed for single‐cell analysis in eukaryotic systems—and many have been systematically benchmarked for multi‐omics integration^104^, due to the fundamental biological differences, it is crucial to evaluate and adapt these approaches using single‐cell multi‐omics data of microbial model organisms before directly applying them on microbial communities. Additionally, temporal lineage tracing^105^ has emerged as a powerful method in single‐cell research and can also provide valuable insights into the division of labor and developmental trajectories within microbial communities. We believe that integrating single‐cell multi‐omics with temporal analysis will significantly advance our understanding of microbial heterogeneity and its impact on human health and disease.
